# Nurses' practice concerning weaning from mechanical ventilation in the intensive care unit

**DOI:** 10.1186/2197-425X-3-S1-A561

**Published:** 2015-10-01

**Authors:** M Borkowska, S Labeau, S Blot

**Affiliations:** Ghent University, Ghent, Belgium; University College Ghent, Ghent, Belgium

## Introduction

Weaning from mechanical ventilation is an important responsibility for ICU nurses [1].

## Objectives

To identify weaning practices among ICU nurses.

## Methods

A cross-sectional, self-administrated survey was developed. Consensus on content validity was achieved through a Delphi procedure among experts. The survey was distributed and collected during the annual congress of the Flemish Society of Critical Care Nurses (Dec. 2014).

## Results

423 attendants completed the survey (response rate: 66%) of which 342 ICU nurses were included for further analysis. These are employed in general (73%) or university hospitals (27%) and are working in mixed (66%), surgical (18%) or medical (13%) ICUs.

Nurses working in university hospitals reported higher availability of weaning protocols (44% vs 29%; p = 0.016). 22% of nurses reported the availability of both weaning and sedation protocol. Protocols are paper-based (23%) or computer-assisted (5%) and in 20% of the cases never used. Nearly 20% of the nurses got some information or training about use of their protocol.

Most frequently chosen weaning modes are CPAP (77%), BiLevel/BIPAP (74%) and PSV (59%). Nurses report to autonomously change ventilation modes (68%), pressure support (PS) level (66%) and respiratory rate (49%). Before starting a spontaneous breathing trial (SBT) evaluation of respiratory status (83%), respiratory capacity (73%) and oxygenation (63%) are considered. Spontaneous breathing on T-tube (58%) is the most frequently chosen SBT approach next to CPAP (57%) and PS ventilation with minimal PS (46%). Duration of first SBT between 30 and 120 minutes was found in nearly 50% of responses but it was not associated with the presence or absence of a weaning protocol (p = 0.57). Less than 30 minutes SBT was reported by 45% of nurses. In majority of the cases is SBT repeated 3 times per day (38%) and mostly at the daytime (99% vs. 37%).

SBT is considered successful if adequate gas exchange is maintained during the procedure (89%), and prematurely terminated in case of signs of exhaustion (89%), inadequacy of gas exchange (90%) or hemodynamic instability (66%). Prior to extubation almost all nurses (94%) indicate oxygenation as an important parameter.

When it comes to weaning, nurses are generally satisfied about the cooperation with MDs. Decision making in weaning is mostly taken by MDs (91%).

## Conclusions

Less than half of nurses use weaning protocols. Variability in nurses' practical approaches to weaning appears to be considerable. Physicians are mostly involved in decision making on weaning.
